# A multichannel MEG time–frequency analysis framework for detecting stage -specific effects of spatial distraction in visual-spatial working memory

**DOI:** 10.3389/fnins.2026.1844642

**Published:** 2026-05-08

**Authors:** Zhengchen Li, Qian Liang, Wuqiang Xiao, Tao Li, Zhilin Chen, Xiaoshun Tang, Yetong Ouyang, Zhexue Huang, Limin Sun, Xiaohui Tang, Xijin Wang

**Affiliations:** 1 Department of Neurology, Tongji Hospital, School of Medicine, Tongji University, Shanghai, China; 2 Department of Internal Emergency Medicine, Tongji Hospital, School of Medicine, Tongji University, Shanghai, China; 3 State Key Laboratory of Functional Materials of Informatics, CAS Center for Excellence in Superconducting Electronics (CENSE), Shanghai Institute of Microsystem and Information Technology (SIMIT), Chinese Academy of Sciences, Shanghai, China

**Keywords:** cognitive control, inhibitory control, magnetoencephalography (MEG), oscillatory dynamics, visual-spatial working memory

## Abstract

**Introduction:**

Spatial distraction can disrupt visual-spatial working memory (VSWM), but its stage-dependent effects on multichannel neural dynamics remain insufficiently characterized. This study presents a multichannel magnetoencephalography (MEG) time—frequency analysis framework to detect stage-specific oscillatory responses to spatial distraction during a VSWM task.

**Methods:**

MEG signals were recorded from healthy participants under Distractor and No-distractor conditions and analyzed across encoding, maintenance, and retrieval/decision epochs. Time–frequency power was estimated in the delta, theta, alpha, beta, and gamma bands, and condition differences were evaluated using sensor-level spatiotemporal cluster-based permutation testing and Bonferroni correction within each predefined epoch.

**Results:**

The proposed analysis revealed a clear stage-specific pattern, with the most prominent modulation occurring during maintenance. Specifically, distraction induced robust and sustained increases in theta-, alpha-, and beta-band power during the retention interval (all cluster-level *p* < 0.01). Theta activity increased rapidly after maintenance onset and remained elevated throughout the full maintenance period over bilateral temporal, and widespread parieto-occipital sensors, while alpha and beta enhancements also showed temporally continuous and spatially stable patterns across widespread sensor networks.

**Discussion:**

These findings highlight sustained large-scale oscillatory modulation as a key neural signature of distraction during mnemonic maintenance. The study provides an interpretable multichannel signal-analysis perspective on distraction effects in working memory and offers a practical framework for stage-resolved analysis of brain dynamics in cognitive tasks.

## 1 Introduction

Working memory is a core cognitive system that temporarily maintains and manipulates task-relevant information to support perception, reasoning, and goal-directed behavior (Baddeley, 1992; Cowan, 2017). Within this system, visual-spatial working memory (VSWM) is especially important because it enables the short-term retention and control of spatial locations and visual configurations that are essential for navigation, attention allocation, and action planning (Lundqvist et al., 2023; McAfoose and Baune, 2009). In natural environments, however, VSWM must operate in the presence of frequent irrelevant stimuli. The ability to resist distraction is therefore fundamental to successful cognition. Understanding how the brain suppresses or accommodates distracting information has become a central question in cognitive neuroscience (Lorenc et al., 2021). Spatial distraction is particularly consequential because it directly competes with the spatial-attentional processes on which VSWM depends (Buschman, 2021). Moreover, distraction may influence different stages of task performance, including cue-related prioritization, mnemonic maintenance, and later decision processes (Desimone and Duncan, 1995; Feldmann-Wustefeld and Vogel, 2019; McNab and Dolan, 2014; Rutman et al., 2010; Zanto and Gazzaley, 2009). Clarifying when and how these effects arise requires neural measures with high temporal precision.

Previous studies have shown that neural oscillations provide a powerful framework for characterizing working memory and distraction. Alpha activity has been linked to selective inhibition and the protection of task-relevant representations, whereas beta oscillations are often associated with top-down control, maintenance of cognitive states, and large-scale coordination across cortical systems (Ashton et al., 2023; Chen and Huang, 2015; Clayton et al., 2018; Engel and Fries, 2010; Fries, 2015; Wilken et al., 2023; Woodman et al., 2022). Theta and delta rhythms have likewise been implicated in executive control, sequencing, and sustained cognitive engagement (Sevim et al., 2025; Williams et al., 2019). In parallel, magnetoencephalography (MEG) offers an ideal tool for examining these processes because of its millisecond temporal resolution and sensitivity to distributed cortical dynamics (Gross, 2019). Nevertheless, many studies focus on only one stage of the task, most commonly the retention interval, making it difficult to determine whether distractor-related effects originate during cue processing, maintenance, or response selection (Ashton et al., 2023; Chen and Huang, 2015; Matsuoka et al., 2020; Wianda and Ross, 2019). In addition, prior findings across frequency bands are often heterogeneous, and the temporal evolution as well as topographic organization of distractor-related oscillatory responses remains insufficiently resolved (Ferrante et al., 2026; Hall et al., 2024; Jacob et al., 2018; Jung et al., 2025; Sattelberger et al., 2024). As a result, it is essential to provide a stage-specific description of how spatial distraction modulates oscillatory dynamics throughout the processing of VSWM.

To address these gaps, the present study investigated the stage-specific neural signatures of spatial distraction during a VSWM task using MEG time-frequency analysis. We divided the task into three predefined epochs—encoding, maintenance, and retrieval/decision—and examined oscillatory power in five canonical frequency bands: delta, theta, alpha, beta, and gamma. By directly contrasting Distractor and NoDistractor conditions, we aimed to determine whether the neural impact of distraction is stage-specific, frequency-specific, and topographically organized across the task (Beffara et al., 2022; Bonnefond and Jensen, 2012; Magosso and Borra, 2024; Solis-Vivanco et al., 2021; Zeng et al., 2024; Zhou et al., 2023). In this way, our study aims to offer a tentative, time-resolved perspective on how distractors may interact with VSWM throughout different cognitive stages.

The main contributions of this study are threefold. First, we present a stage-specific analytical framework to investigate the oscillatory effects of spatial distraction across encoding, maintenance, and retrieval/decision in VSWM. Second, we show that these effects are stage-dependent, with the strongest and most sustained modulation occurring during maintenance in the theta, alpha, and beta bands. Third, by characterizing the temporal continuity and sensor-level spatial organization of these effects, our findings suggest that distraction during VSWM maintenance is accompanied by sustained and distributed oscillatory dynamics.

## 2 Materials and methods

### 2.1 Participants

Twenty-two healthy volunteers were initially recruited for this study. After quality control, two participants were excluded, one because of excessive artifacts in the MEG recordings and the other because of missing structural MRI data. Consequently, data from 20 right-handed participants were retained for the final analysis. All participants had normal or corrected-to-normal vision and provided written informed consent before the experiment. The study protocol was approved by the Ethics Committee of Shanghai Tongji Hospital.

### 2.2 Experimental design and task procedure

**FIGURE 1:**
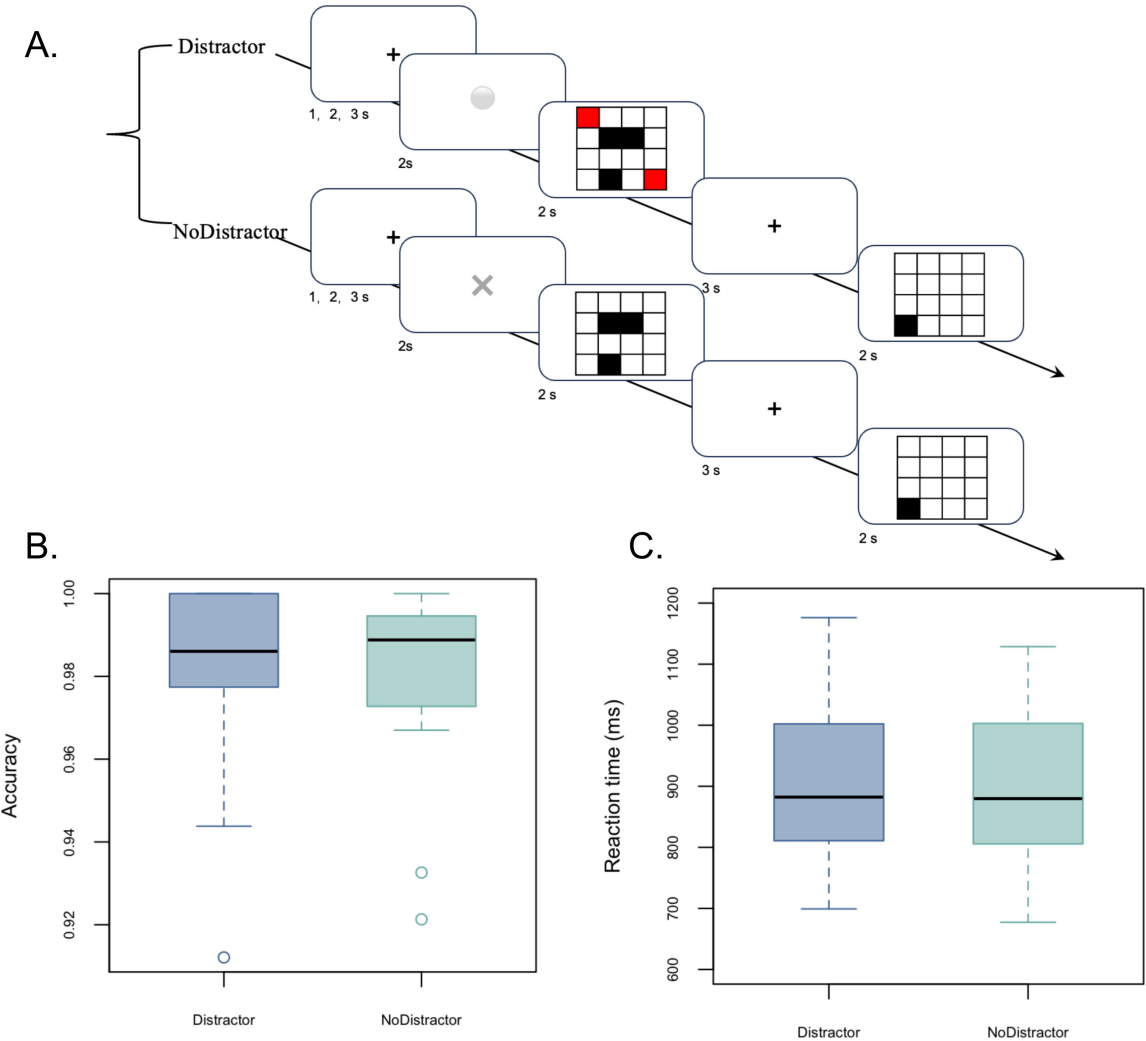
Schematic illustration of the cue-guided visual-spatial working memory task under distractor and no-distractor conditions and behavioral performance of participants. **(A)** Participants were asked to remember the three black squares (targets) and two red squares (distractors, which appeared randomly) during the maintenance period. Following delay, a black probe square was presented, and they were asked to response whether its location matched that of any of the three black target squares. Accuracy **(B)** and reaction time **(C)** did not statistically differ between conditions.

To investigate stage-specific neural responses to spatial distraction during VSWM, participants performed a VSWM task comprising distractor and no-distractor conditions, as illustrated in Figure 1. Each trial began with a central fixation cross presented for a jittered interval to reduce temporal expectancy. This was followed by a symbolic cue. A cue marked by “×” indicated an upcoming no-distractor trial, whereas a cue marked by “O” indicated an upcoming distractor trial. The two cue types occurred with equal probability and were presented in a fixed counterbalanced order across participants.

After the cue period, a memory array was displayed on a 4 × 4 spatial grid. In the No-distractor condition, the array contained three black squares corresponding to target items. In the distractor condition, the array contained the same three black target squares together with two red distractor squares. All items were positioned at random non-overlapping locations within the grid. After stimulus offset, participants maintained the target locations across a retention interval. A probe array was then presented, containing a single black square located at one grid position. Participants were instructed to determine whether the probe location matched any of the previously presented target locations and responded with a left mouse click for “yes” and a right mouse click for “no.”

Before formal data collection, participants completed a brief practice session to ensure that the task instructions were fully understood. The formal experiment consisted of 180 trials divided into six blocks. Stimulus presentation and response recording were controlled using E-Prime software (version 1.2; Psychology Software Tools, Inc., Sharpsburg, PA, United States), and all visual stimuli were displayed on a projection screen.

### 2.3 MRI acquisition

To support MEG–MRI coregistration and source-space reconstruction, a high-resolution T1-weighted anatomical MRI scan was acquired for each participant using a Philips 3.0∼T scanner. The acquisition parameters were as follows: repetition time (TR) = 2,530 ms, echo time (TE) = 2.98 ms, inversion time (TI) = 1,100 ms; flip angle = 7°; slice thickness = 1 mm; matrix size = 256 × 224; and field of view (FOV) = 25.6 cm.

### 2.4 MEG acquisition

Neuromagnetic activity was recorded using a 306-channel TRIUX neo MEG system (MEGIN, Finland), comprising 102 magnetometers and 204 planar gradiometers, in a magnetically shielded room at the Institute of Neuroscience, Center for Excellence in Brain Science and Intelligence Technology, Chinese Academy of Sciences, Shanghai, China. MEG data were continuously acquired while participants performed the VSWM task. Prior to recording, the head shape and the three fiducial landmarks (nasion, left preauricular point, and right preauricular point) were digitized using a FASTRAK 3D digitizer (Polhemus, Colchester, VT, United States). These landmarks were subsequently used for MEG–MRI coregistration. Electrooculogram (EOG) signals were simultaneously recorded to facilitate the identification and removal of ocular artifacts.

### 2.5 MEG preprocessing

**FIGURE 2:**
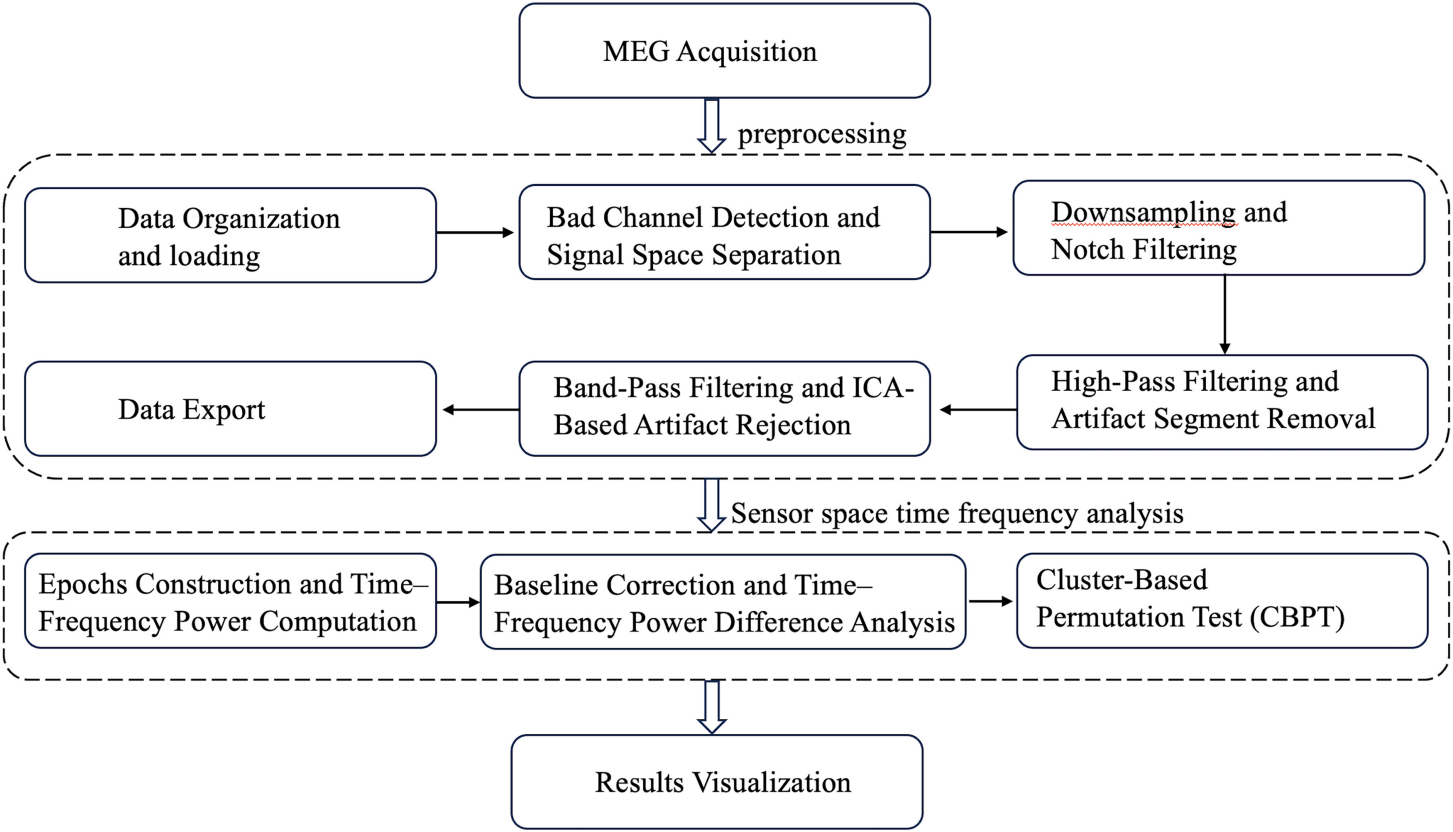
Overall preprocessing and analysis pipeline for the MEG data, including artifact suppression, time–frequency decomposition, source-space reconstruction, and statistical testing.

The overall MEG analysis pipeline is summarized in Figure 2. MEG preprocessing was carried out using the MNE-Python software package. The overall goal of preprocessing was to suppress external interference, remove physiological and non-physiological artifacts, and obtain clean sensor-level signals for subsequent time–frequency and statistical analyses.

First, all automatically detected noisy segments were visually inspected by an experienced MEG analyst to verify the correctness of artifact labeling and to correct possible misclassifications. Next, spatiotemporal signal space separation (SSS) was applied to suppress environmental interference and compensate for small head movements (Taulu et al., 2004; Taulu and Simola, 2006). In the SSS framework, the multichannel MEG measurement vector φ is modeled as the sum of contributions from sources inside and outside the sensor helmet:


φ=Sx=[Si⁢n⁢So⁢u⁢t]⁢[xi⁢nxo⁢u⁢t],

Where S_in_ and S_out_denote the basis matrices associated with internal sources and external source space, respectively, and *x*_*in*_and *x*_*out*_ are the corresponding expansion coefficients. The coefficients are estimated as


x^=[x^i⁢nx^o⁢u⁢t]=S+⁢φ,

Where *S*^+^ denotes the Moore–Penrose pseudoinverse of the combined basis matrix *S* = [*S*_*in*_*S*_*out*_]. The internal and external components are then reconstructed as:


$${\hat{\varphi }}_{i\,n}=S_{i\,n}\,{\hat{x}}_{i\,n}$$

and


$${\hat{\varphi }}_{o\,u\,t}=S_{o\,u\,t}\,{\hat{x}}_{o\,u\,t}$$

After SSS, the data were downsampled to 240 Hz to reduce computational cost while preserving the spectral content relevant to the present analysis. A notch filter was then applied at 50 and 100 Hz to remove line noise and its first harmonic. To further reduce muscle contamination, the annotate_muscle_z score procedure in MNE-Python was used to identify high-frequency transient artifacts on magnetometer channels after high-pass filtering above 90 Hz.

The data were subsequently band-pass filtered between 1 and 90 Hz to suppress slow drifts and high-frequency noise. Independent component analysis (ICA) was then performed to isolate stereotypical artifacts such as eye movements and residual physiological noise. Specifically, FastICA was applied after centering and whitening the data. The observed multichannel signal *x* was modeled as a linear mixture of statistically independent latent sources:


x=A⋅s

Where *A* is the unknown mixing matrix and *s* denotes the vector of independent source components (Hyvarinen and Oja, 2000). The number of components was fixed at 30, and a fixed random seed was used to ensure reproducibility. After ICA decomposition, artifact-related components were identified and removed. Because the present study focused on robust sensor-level oscillatory effects, all subsequent analyses were performed on magnetometer channels only.

### 2.6 Sensor-level time-frequency analysis

To quantify oscillatory responses associated with spatial distraction, sensor-level time–frequency analysis was performed on the preprocessed MEG data. Oscillatory power was evaluated in five canonical frequency bands: delta (1–4 Hz), theta (4–8 Hz), alpha (8–13 Hz), beta (13–30 Hz), and gamma (30–80 Hz).

To capture stage-dependent neural dynamics, each trial was segmented into three task-defined epochs: encoding stage, maintenance stage, and retrieval/decision stage. The encoding stage was intended to capture the encoding of the visuospatial memory array, attentional allocation to the task-relevant display, and the initial formation of working-memory representations; the maintenance stage was designed to isolate active working-memory maintenance and resistance to distraction during the delay interval; and the retrieval/decision stage was defined to reflect probe evaluation, mnemonic comparison, decision formation, and response selection. For each epoch, data were extracted relative to the corresponding event onset, and baseline correction was applied using the pre-stimulus interval. Epochs marked as artifactual during preprocessing were excluded. The remaining trials were then separated according to Distractor condition, yielding one set of epochs for the distractor trials and another for the No-distractor trials.

Time–frequency power was estimated using Morlet wavelet convolution as implemented in MNE-Python (tfr_morlet). The Morlet mother wavelet is defined as


$$\psi \,\left(t\right)={\left(\psi^{2}\right)}^{-1/4}\,e^{i\,2\,\pi \,f_{0}\,t}\,e^{-t^{2}/\left(2\,\sigma^{2}\right)},$$

where *f_0_* denotes the central frequency (set to 1 by default in the normalized mother wavelet) and σ controls the width of the Gaussian envelope. The relationship between the number of cycles (*n*_*cycles*_) and the Gaussian standard deviation for a given frequency of interest *f* is given by:


$$\sigma =\frac{n_{\text{cycles}}}{2\,\pi \,f}$$

Time–frequency power was computed from 1 to 80 Hz in 1-Hz steps, with *n*_cycles_ = *f*/2. This yielded a constant temporal width of


$$\sigma =\frac{1}{4\,\pi }\,s$$

across frequencies. Power estimates were averaged across epochs (average = True), inter-trial coherence was not computed (return_itc = False), and the output was decimated by a factor of 4 after convolution (decim = 4).

### 2.7 Supplementary source-space visualization

To complement sensor-level analysis with anatomically informed source modeling, each participant’s structural MRI data was processed using FreeSurfer for cortical surface reconstruction and anatomical preprocessing. These individualized anatomical reconstructions were then used to establish the correspondence between MEG sensor coordinates and MRI space through rigid-body coregistration based on the digitized head-shape points and head position indicator measurements. Therefore, the source-space maps served as complementary anatomical illustrations of those sensor-level findings.

### 2.8 Statistical analysis

Statistical analysis was designed to identify reliable spatiotemporal differences in oscillatory power between the Distractor and No-distractor conditions. Sensor adjacency was defined according to the physical sensor layout using the mne.channels.find_ch_adjacency function in MNE-Python.

Within each canonical frequency band, time-frequency power was averaged across frequencies, yielding a three-dimensional array (*n*_subjects_ × *n*_times_ × *n*_channels_). For each predefined task epoch and frequency band, distractor-related effects were assessed using sensor-level spatiotemporal cluster-based permutation testing (CBPT), which controls for multiple comparisons while preserving the spatiotemporal dependency structure of MEG data. Specifically, within-subject difference maps (Distractor—No-distractor) were computed and entered into a one-sample spatiotemporal cluster-based permutation test. Candidate clusters were defined as groups of adjacent sensor-time samples exceeding a predefined *t*-statistic threshold (*t*_*threshold*_). Spatial adjacency was determined from the sensor adjacency matrix, and temporal adjacency was defined across consecutive time points. Cluster-level significance was evaluated against a permutation-based null distribution constructed from 1,000 random permutations.

Because the task epochs were predefined by the experimental design and corresponded to distinct processing stages, frequency-band analyses were organized and interpreted separately within each epoch. Within each epoch, five canonical frequency bands were tested. Therefore, in addition to the within-analysis multiple-comparison control provided by CBPT, an additional Bonferroni correction was applied across the five frequency-band analyses within the same epoch, resulting in a corrected significance threshold of 0.05/5 = 0.01. Effects surviving this threshold were treated as corrected significant findings in the main results, whereas effects meeting the original cluster-level criterion (*p* = 0.05) but not the Bonferroni-adjusted threshold were retained only as exploratory findings.

A *post-hoc* sensitivity analysis was conducted using G*Power 3.1 to evaluate whether the present sample size was sufficient to detect meaningful within-subject effects. Because the main inferential analysis was based on subject-level contrast maps entered into a one-sample spatiotemporal cluster-based permutation test, the sensitivity analysis was performed on the corresponding subject-level contrast values. For α = 0.05, power = 0.80, and *N* = 20, the minimum detectable effect size was approximately *d*_*z*_ = 0.66, indicating that the present sample was sufficiently sensitive to detect medium-to-large effects (Gresch et al., 2024; Onishi and Yokosawa, 2023). For descriptive purposes, effect sizes were additionally estimated for all reported significant clusters. Specifically, cluster-averaged within-subject difference values were extracted at the participant level, and Cohen’s *d_z_*was computed to characterize the magnitude of the observed effects (Supplementary Table 1).

Behavioral data were analyzed separately using two-tailed paired-samples *t*-tests. Continuous variables are reported as mean ± standard deviation. Behavioral analyses were performed in R version 4.5.1 (R Foundation for Statistical Computing, Vienna, Austria).

## 3 Results

### 3.1 Behavioral analysis

Of the 22 enrollees, two participants were excluded due to excessive artifacts in their MEG data (*n* = 1) and missing MRI data (*n* = 1). The remaining 20 participants (mean age: 26 years, SD: 2.90, range: 21–30 years, female: 12) were included in the final analyses (Table 1). They performed well on the VSWM task, responding accurately on 98.10% (SD = 2.18%) of all trials, with no significant difference in accuracy between the Distractor and No-distractor trials [98.11% ± 2.09% vs. 98.08% ± 2.28%, *t*(19) = 0.153, *p* = 0.880, Figure 1B]. Likewise, reaction time did not differ significantly between the Distractor and No-distractor conditions [(891.00 ± 119.05 ms vs. 906.82 ± 122.41 ms, *t*(19) = –1.032, *p* = 0.315, Figure 1C].

**TABLE 1:** Demographic and behavioral data for participants.

Variables	No-distractor	Distractor	*T*-value	*P*-value
Age, y	26.0 ± 2.90	26.0 ± 2.90	/	/
Male, No. (%)	40.0%	40.0%	/	/
Education, y	18.5 ± 1.4	18.5 ± 1.4	/	/
Accuracy	98.11% ± 2.09%	98.08% ± 2.28%	0.153	0.880
Reaction time, ms	891.00 ± 119.05	906.82 ± 122.41	-1.032	0.315

Results expressed as mean scores ± standard deviation (normally distributed data).

### 3.2 MEG time-frequency responses and cluster-based statistics

Time—frequency responses were compared between the two conditions (Distractor vs. No-distractor) using cluster-based permutation tests across sensors and time, performed separately for delta, theta, alpha, beta, and gamma bands. For each significant cluster, we report the cluster-level *p*-value before and after Bonferroni correction, together with the corresponding time range and main sensor topography.

#### 3.2.1 Maintenance stage (0–3 s): distractor induces sustained oscillatory enhancement across frequency bands

During the maintenance stage, spatial distraction was associated with sustained increases in oscillatory activity, with robust effects observed in the theta, alpha, and beta bands. These three band-specific effects remained statistically significant both before and after Bonferroni correction, indicating reliable and temporally sustained distractor-related modulation throughout maintenance. In addition, a delta-band effect was observed before correction, but it did not survive Bonferroni correction (Matsuoka et al., 2020; Wianda and Ross, 2019).

##### 3.2.1.1 Theta band (4–8 Hz): rapid-onset and widespread sustained oscillatory enhancement

**FIGURE 3:**
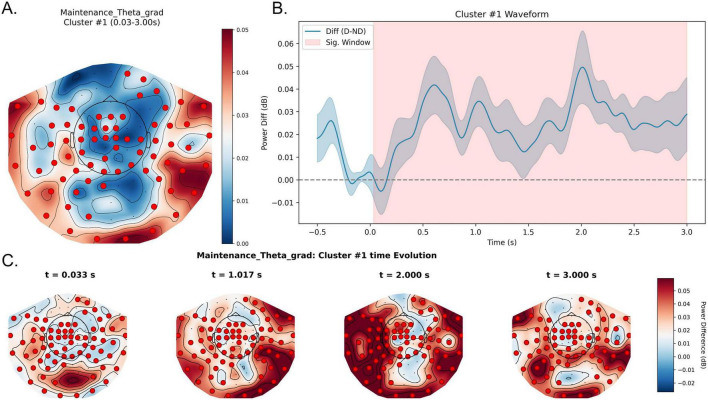
Sustained theta-band enhancement during the maintenance stage under distraction. **(A)** Topography of the maintenance theta effect in the significant window (0.03–3.00 s). Colors denote Distractor—No-distractor (dB), and red markers indicate sensors contributing to the significant spatiotemporal cluster. **(B)** Waveform of the theta power difference averaged across significant sensors, with the shaded region marking 0.03–3.00 s (throughout retention). Positive values indicate higher theta power in the Distractor condition relative to the No-distractor condition. **(C)** Topographic snapshots of Distractor—No-distractor at representative time points spanning the significant window, illustrating the rapid onset and sustained spatial configuration of the effect. D, Distractor; ND, No-distractor.

In the theta band, a highly significant positive spatiotemporal cluster was observed (cluster-level *p* < 0.01), spanning approximately 0.03–3.00 s relative to maintenance onset, almost covering the entire maintenance period. Waveform analyses showed consistently higher theta power under distraction, indicating sustained enhancement of theta-band activity throughout maintenance. The spatial distribution formed a broad network involving frontal, bilateral temporal, and widespread parieto-occipital sensors. This distractor-induced increase in theta power emerged rapidly within a very short latency after maintenance onset (approximately 30 ms) and remained a highly stable, brain-wide network activation pattern throughout the 3 s maintenance period (Figure 3).

##### 3.2.1.2 Alpha band (8–13 Hz): sustained parieto-occipital/temporal oscillatory enhancement

**FIGURE 4:**
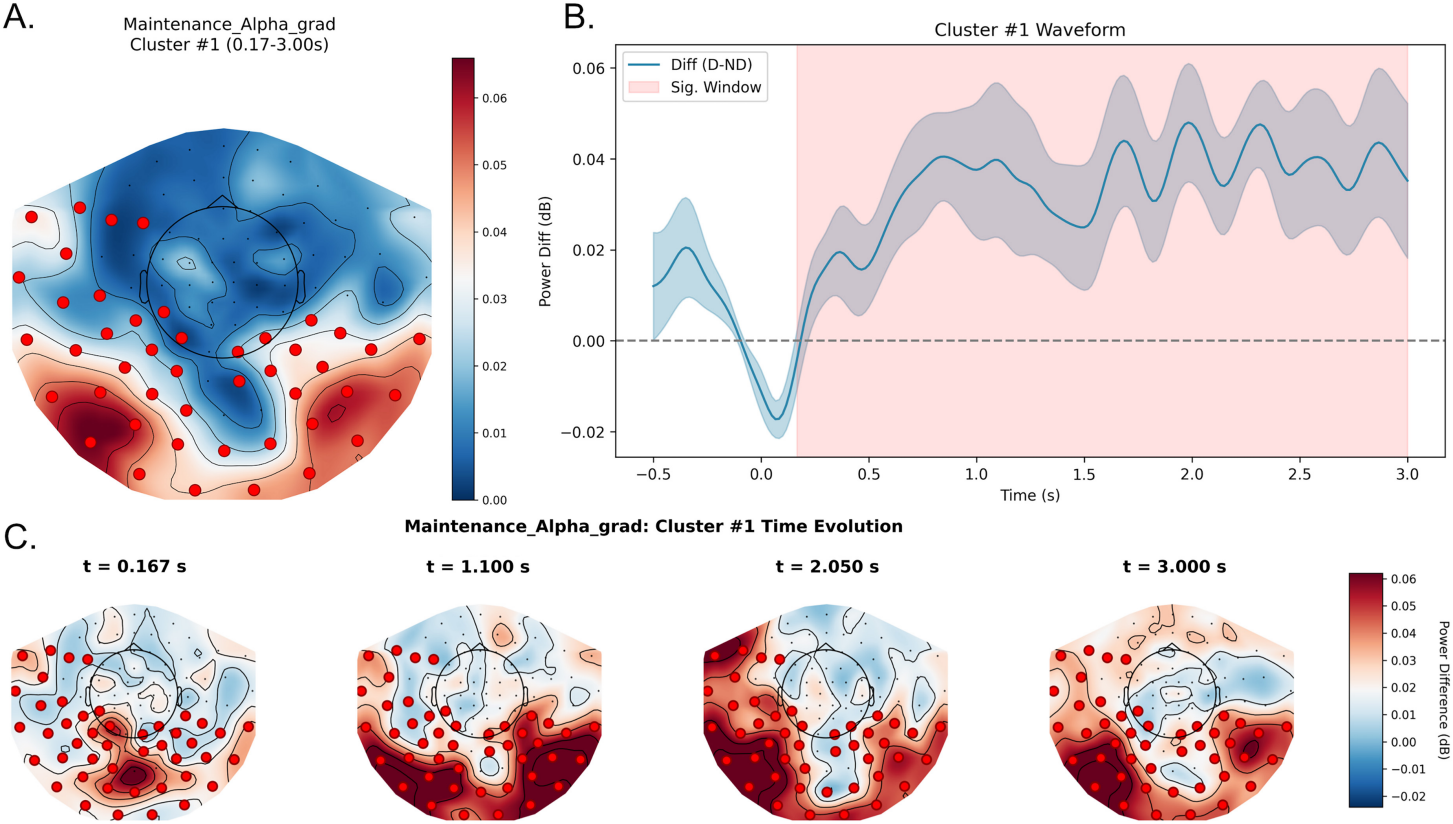
Sustained alpha-band enhancement during the maintenance stage under distraction. **(A)** Topography of the maintenance alpha effect in the significant window (0.17–3.00 s). Colors denote Distractor—No-distractor (dB), and red markers indicate sensors contributing to the significant spatiotemporal cluster, which shows a posteriorly distribution. **(B)** Waveform of the alpha power difference averaged across significant sensors, with the shaded region marking 0.17–3.00 s (almost throughout retention). Positive values indicate higher alpha power in the Distractor condition relative to the No-distractor condition. **(C)** Topographic snapshots of Distractor—No-distractor at representative time points spanning the significant window, illustrating the sustained spatial configuration of the effect. D, Distractor; ND, No-distractor.

In the alpha band, we identified a highly significant positive spatiotemporal cluster (cluster-level *p* < 0.01) spanning approximately 0.17–3.00 s relative to maintenance onset. Waveform analyses showed reliably higher alpha-band power in the distractor condition, indicating enhancement of alpha-band activity during maintenance. Significant sensors concentrated over bilateral parieto-occipital and temporal regions, with a slightly broader distribution over the left hemisphere (Figure 4). Time-resolved maps indicated that the distraction-related alpha enhancement was highly temporally continuous and spatially stable across the maintenance interval.

##### 3.2.1.3 Beta band (13–30 Hz): early-onset and sustained central/right parieto-occipital oscillatory enhancement

**FIGURE 5:**
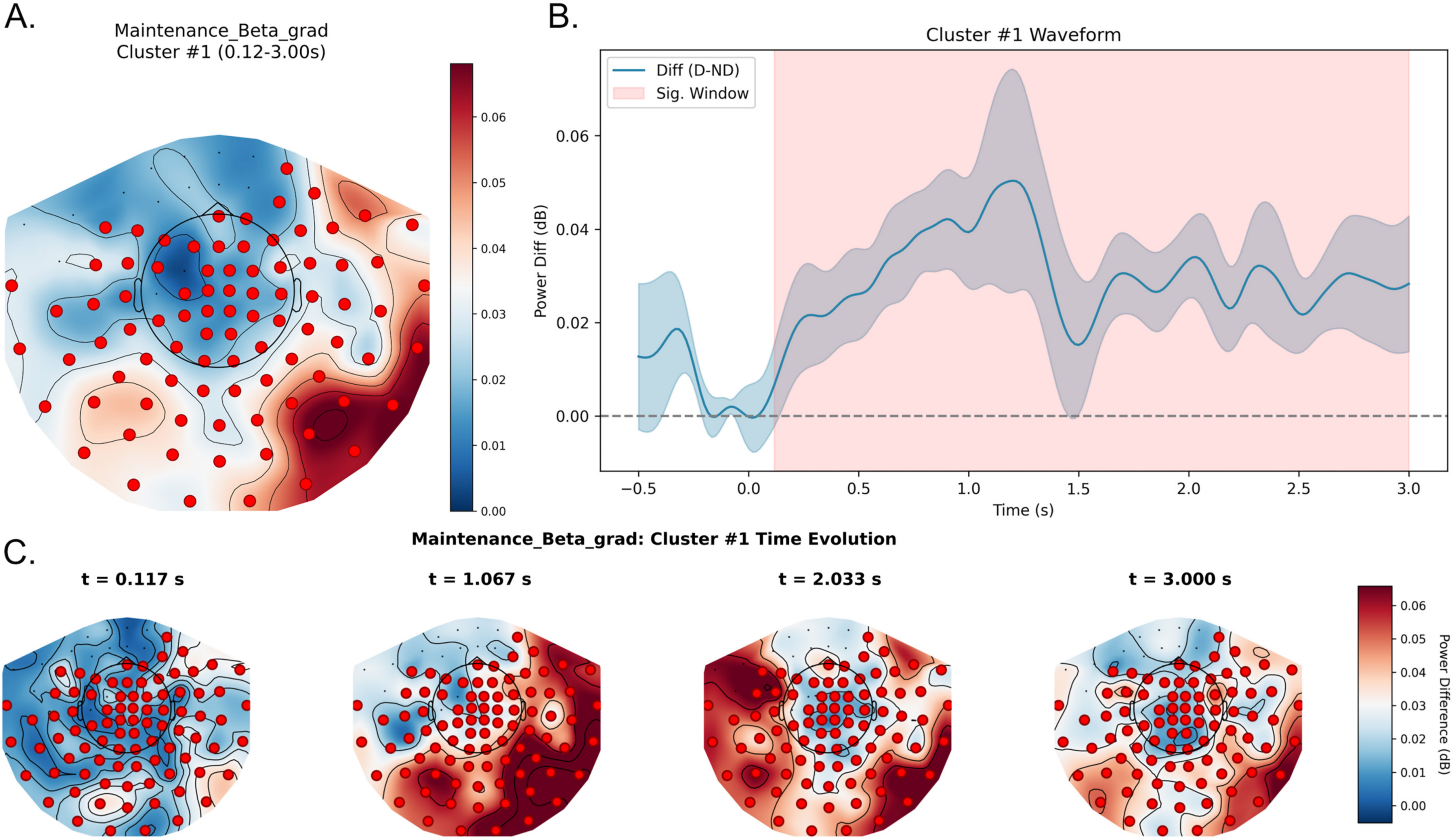
Sustained beta-band enhancement during the maintenance stage under distraction**. (A)** Topography of the maintenance beta effect in the significant window (0.12–3.00 s), showing a central–parieto-occipital significant sensor pattern across retention. Colors denote Distractor—No-distractor (dB), and red markers indicate sensors contributing to the significant spatiotemporal cluster. **(B)** Waveform of the beta power difference averaged across significant sensors, with the shaded region marking 0.12–3.00 s (almost throughout retention). Positive values indicate higher beta power in the Distractor condition relative to the No-distractor condition. **(C)** Topographic snapshots of Distractor—No-distractor at representative time points spanning the significant window, illustrating the early onset and sustained spatial configuration of the effect. D, Distractor; ND, No-distractor.

In the beta band, a highly significant positive spatiotemporal cluster (cluster-level *p* < 0.01) was observed, spanning approximately 0.12–3.00 s relative to maintenance onset. Waveform analyses showed higher beta power in the distractor condition, indicating pronounced enhancement of beta-band activity during maintenance. Topographically, significant sensors formed a dense cluster over central regions and extended to the right parieto-occipital and temporal sensors (Figure 5). Time-evolution maps showed that the beta enhancement emerged rapidly (approximately 100 ms) after maintenance onset and maintained a stable spatial pattern across the full maintenance window.

**FIGURE 6:**
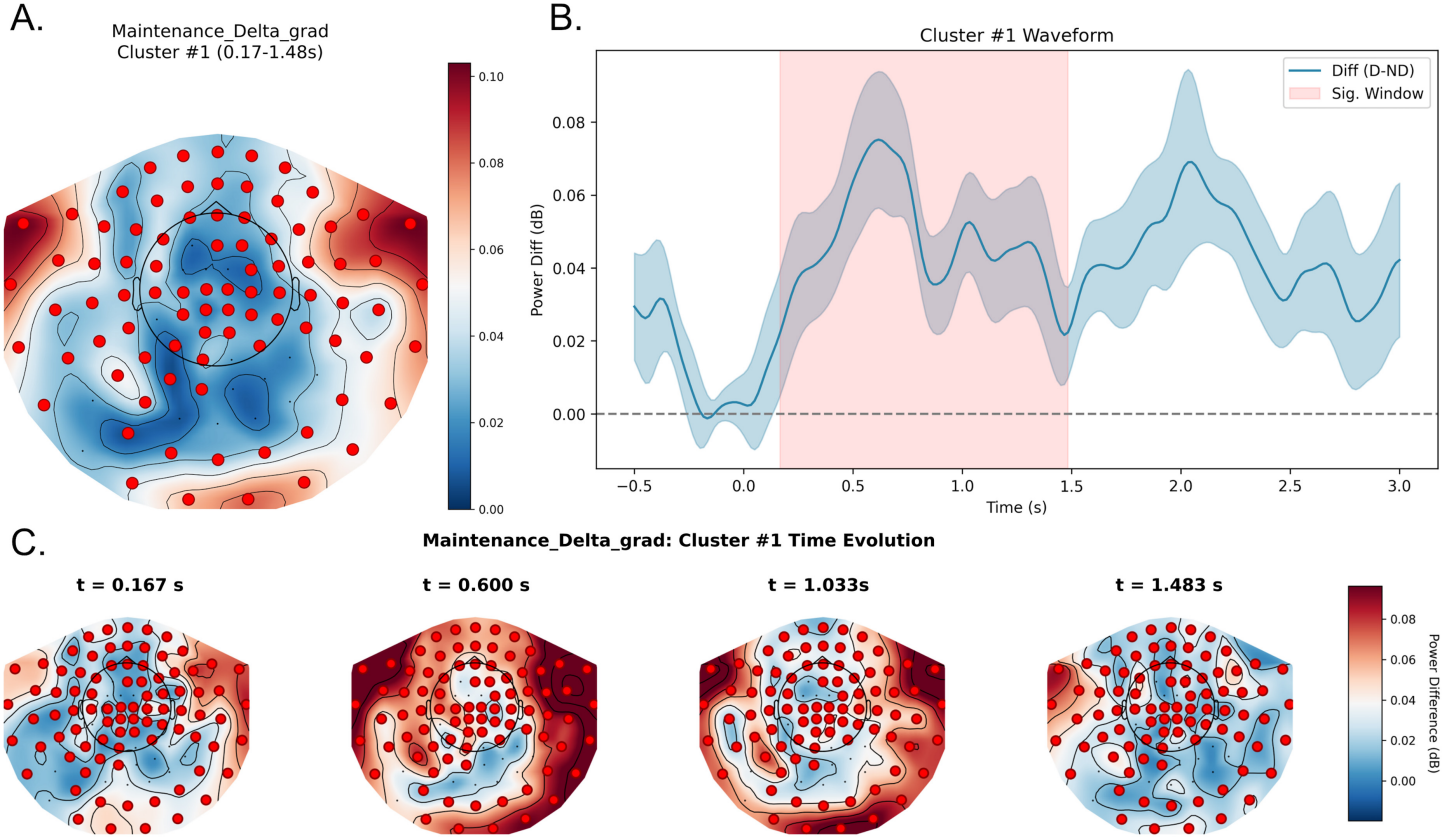
Exploratory early right-lateralized delta-band cluster during the maintenance stage under distraction. **(A)** Topography of the uncorrected delta effect during 0.17–1.48 s after maintenance onset. Colors represent Distractor—No-distractor (dB), and red dots mark sensors included in the cluster. **(B)** Waveform of the delta power difference averaged across cluster sensors, with the shaded region marking 0.17–1.48 s. Positive values indicate higher delta power in the Distractor condition relative to the No-distractor condition. **(C)** Topographic snapshots show an exploratory right-lateralized delta pattern during early maintenance. D, Distractor; ND, No-distractor.

**FIGURE 7:**
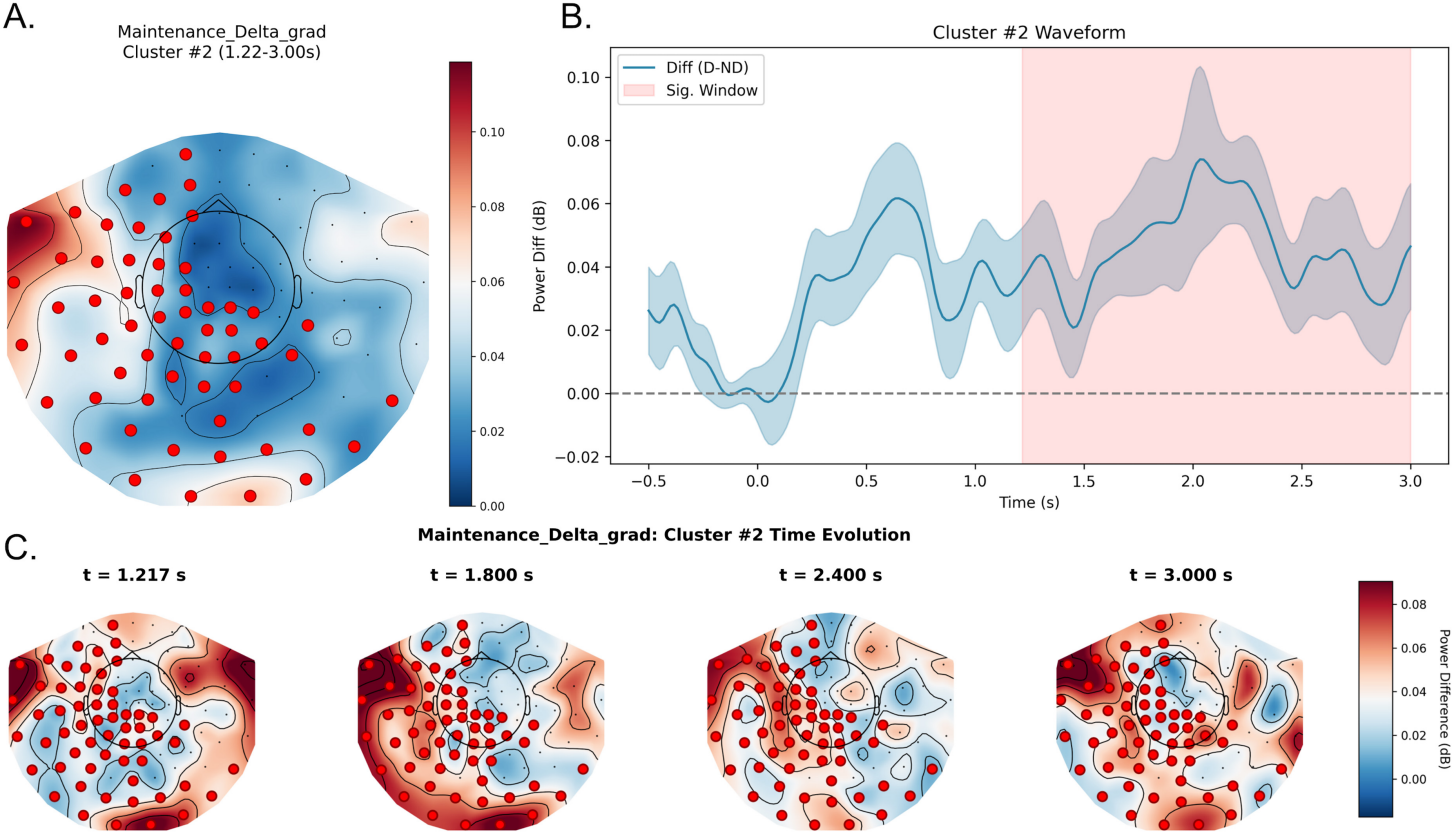
Exploratory later left-lateralized delta-band cluster during the maintenance stage under distraction. **(A)** Topography of the uncorrected delta effect during 1.22–3.00 s after maintenance onset. Colors represent Distractor—No-distractor (dB), and red dots mark sensors included in the cluster. **(B)** Waveform of the delta power difference averaged across cluster sensors, with the shaded region marking 1.22–3.00 s. Positive values indicate higher delta power in the Distractor condition relative to the No-distractor condition. **(C)** Topographic snapshots show an exploratory left-lateralized delta pattern during late maintenance. D, Distractor; ND, NoDistractor.

In the delta band (1–4 Hz), two positive spatiotemporal clusters were identified at the uncorrected level during the maintenance stage (cluster 1: *p* = 0.018; cluster 2: *p* = 0.024). However, neither cluster survived Bonferroni correction and therefore these effects should be interpreted as exploratory. At the descriptive level, waveform analyses suggested enhanced delta activity under distraction throughout maintenance. The first cluster was observed during the early-to-mid portion of the maintenance interval (approximately 0.17–1.48 s) and showed a predominantly right-lateralized distribution over right frontal, right temporal, and right parieto-occipital sensors (Figure 6). The second cluster emerged later (approximately 1.22–3.00 s) and exhibited a more left-lateralized pattern involving left frontal, left central, left temporal, and portions of the left parietal regions (Figure 7). These uncorrected patterns may suggest a temporal transition from right-dominant to left-dominant delta activity during maintenance, although this observation did not remain significant after multiple-comparison correction.

No significant spatiotemporal clusters were detected in the gamma band during maintenance stage (all cluster-level *p* > 0.05).

#### 3.2.2 Encoding stage (0–2 s) and retrieval/decision epoch (0–2 s): no significant distraction-related clusters across frequency bands

**FIGURE 8:**
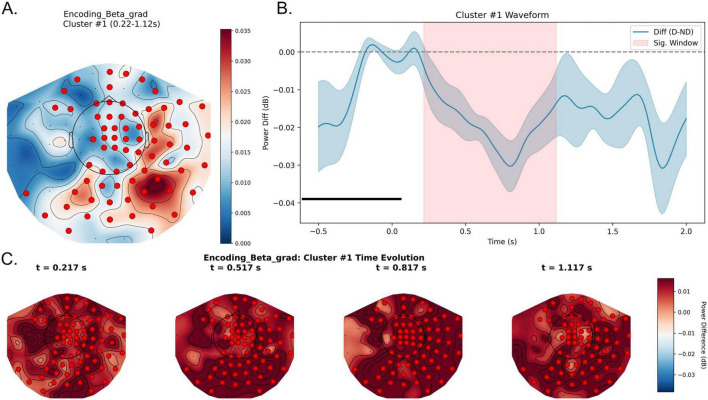
Exploratory beta-band modulation during the encoding epoch under distraction. **(A)** Topographic map of the uncorrected CueOn beta-band cluster (0.22–1.12 s). Colors denote the power difference (Distractor—No-distractor, dB). Red dots indicate sensors contributing to the uncorrected cluster. **(B)** Waveform of the beta power difference averaged across cluster sensors, with the shaded region marking 0.22–1.12 s. Negative values indicate lower beta power in the Distractor condition, consistent with beta suppression under distraction. **(C)** Topographic snapshots show an exploratory beta pattern distributed mainly over central and right parieto-occipital regions. D, Distractor; ND, No-distractor.

In the encoding stage, a beta-band spatiotemporal cluster was observed at the uncorrected level (cluster-level *p* = 0.031, Distractor < No-distractor), indicating beta suppression under distraction. This effect extended from approximately 0.22 to 1.12 s after encoding onset, with significant sensors mainly distributed over central and right parieto-occipital regions, along with additional temporal involvement (Figure 8). However, this cluster did not survive Bonferroni correction and is therefore reported as an exploratory finding. No significant clusters were observed in the delta, theta, alpha, or gamma bands.

In the retrieval/decision epoch, we applied the same cluster-based permutation analysis to the delta, theta, alpha, beta, and gamma bands, and no significant spatiotemporal clusters were observed. Thus, distraction-related oscillatory differences were confined to the maintenance epoch, and no band-limited effect was detected during encoding and retrieval/decision in the present analysis.

## 4 Discussion

In the current study, we used MEG time-frequency analyses to investigate how spatial distraction modulates oscillatory activity across three task-defined epochs: encoding, maintenance, and retrieval/decision. Although significant MEG power differences were observed during the Maintenance stage, no significant differences were found in reaction time and accuracy between Distraction and No-distraction conditions. Given that our participants were young and highly educated, this result may suggest relatively preserved resistance to distraction or efficient compensatory processing. Therefore, the effect of spatial distraction in the present study appears to be reflected primarily in stage-specific neural modulation rather than in overt behavioral impairment. During maintenance, distraction induced robust and sustained increases in theta-, alpha-, and beta-band power. In contrast, no distractor-related clusters survived Bonferroni correction in the encoding or retrieval/decision epochs, although an uncorrected beta-band effect was observed during encoding. Together, these findings suggest that spatial distraction modulates oscillatory dynamics primarily during the maintenance stage of VSWM.

The most robust finding of the present study was the sustained enhancement of theta-, alpha-, and beta-band activity during the maintenance stage under distraction. Rather than showing a brief or isolated response, spatial distraction was associated with temporally continuous and spatially distributed oscillatory modulation throughout the retention interval, suggesting that coping with interference in VSWM relies on prolonged neural engagement during mnemonic maintenance.

The enhancement of theta emerged with very short latency after maintenance onset and remained widespread across frontal, temporal, and parieto-occipital sensors. This rapid and distributed theta enhancement reflects a rapid and sustained global response to external spatial interference. This result is consistent with increased engagement of a global control mode under distraction (Asanowicz et al., 2023). Theta-band synchronization over frontal networks is thought to reflect strong engagement of the central executive system and top-down cognitive control. In the presence of spatial distraction, the brain may increase the control load of working memory to sustain attentional allocation through enhanced theta oscillations, coordinating large-scale neural networks. Consequently, task-relevant representations can be successfully maintained under strong interference (Magosso and Borra, 2024; Pastotter et al., 2024; Surrey et al., 2025; Tian et al., 2025).

The sustained enhancement of alpha-band activity suggests that distraction increases the need for attentional shielding and selective suppression of task-irrelevant processing during maintenance. Our results showed that these effects spread over bilateral parieto-occipital and temporal sensors, which is consistent with the visual-parietal circuits that support spatial attention (Bonnefond and Jensen, 2012; Magosso and Borra, 2024; Zeng et al., 2024; Zhou et al., 2023). To protect ongoing cognitive representations from external spatial distraction, the brain may engage top-down control to upregulate alpha oscillations in sensory and related cortices (Zhou et al., 2023). This result reflects an active inhibitory mechanism in the cortex (Bonnefond and Jensen, 2025).

During the maintenance phase of working memory, spatial distraction not only modulated alpha-band activity but also produced enhancement of beta-band oscillations (Liljefors et al., 2024). The sustained enhancement of beta-band activity reflects strong maintenance of the current cognitive state (the “status quo”) and top-down executive control (de Vries et al., 2019; Jung et al., 2025; Lorenc et al., 2021). In the presence of strong external distraction, we observed increased beta activity over central and parieto-occipital regions. This activity may index an active consolidation of sensorimotor and working-memory representations. Such a process could enhance internal network rigidity, helping to resist disruption of the maintained memory goal by spatial distraction (Morton and Polyn, 2017).

Taken together, these findings suggest that distraction during working-memory maintenance does not simply induce a global increase in oscillatory power, but rather engages multiple temporally sustained and spatially distributed control-related processes (Ericson et al., 2025). In the present data, theta enhancement may be more closely related to large-scale coordination and sustained control demands, alpha enhancement to sensory gating and representational protection, and beta enhancement to top-down filtering and maintenance of the current task state. Nevertheless, these interpretations should remain cautious, because the present analyses were conducted at the sensor level.

Although the encoding beta-band effect and the maintenance delta-band effect did not survive Bonferroni correction, they may still provide exploratory clues about the temporal dynamics of distraction processing in VSWM. The negative beta difference during encoding stage indicates that the distractor condition elicited a stronger beta suppression shortly after encoding onset, and this effect was temporally continuous and spatially stable. The more pronounced and temporally continuous beta suppression under distraction may reflect an extended period of resource allocation to maintain task goals while mitigating the impact of competing spatial inputs (Engel and Fries, 2010; Jung et al., 2025; Sattelberger et al., 2024). In other words, the brain recruits more attentional resources and engages stronger top-down cognitive control to sustain task goals when external interference appears (Ferrante et al., 2026; Hall et al., 2024; Jacob et al., 2018). However, because this effect occurs within the task-defined encoding epoch, it most likely reflects distractor-related modulation of cue processing and attentional orienting. Delta activity exhibited two highly significant positive clusters with a clear temporal sequence and lateralization shift. These results may reflect a sequential cognitive strategy adopted to cope with external interference. The early right-lateralized delta synchronization (0.17–1.48 s) is commonly associated with attentional reorienting and rapid suppressive responses to salient distractors mediated by the right-hemisphere frontoparietal system (Spadone et al., 2021). As time progresses, the later left-lateralized delta synchronization (1.22–3.00 s) may instead reflect a subsequent stage of deeper stabilization of working-memory representations (Slaats et al., 2023). This sustained oscillatory activity concentrated over left fronto-temporo-parietal regions may indicate increased recruitment of inner-speech-based rehearsal or semantic processing networks. Taken together, these preliminary observations raise the possibility that coping with spatial interference may involve different oscillatory operations at different task stages.

During retrieval/decision, no significant clusters were observed across delta, theta, alpha, beta, and gamma, suggesting that distractor-related oscillatory differences were most prominent prior to probe-based decision and response selection. This phase specificity supports the view that distraction mainly perturbs the establishment and stabilization of internal representations during cue processing and maintenance (Magosso and Borra, 2024). However, the null result does not preclude weaker or spatially focal effects during Choice.

There are several limitations to this study. First, the sample size is limited. Although we used cluster-based permutation statistics to control for multiple comparisons and enhance robustness at the group level, a limited sample may increase uncertainty in effect-size estimates. In the future, larger cohorts will be needed to obtain more stable estimates of distractor-related effects across epochs and frequency bands. Second, the present results are based on sensor-level topographies, which can blur anatomical specificity. We are therefore conducting source-space analyses to identify cortical generators in cognitive patients performing VSWM tasks with and without a distractor. More importantly, the coexistence of sustained low-frequency enhancement with alpha and beta motivates follow-up analyses targeting network interactions.

## 5 Conclusion

This study shows that spatial distraction modulates visual-spatial working memory in a stage-specific manner. The results suggest that distractor resistance in visual-spatial working memory is supported by dynamic and temporally organized oscillatory mechanisms rather than a single uniform process. Specifically, distraction was associated with strong and sustained enhancement in the theta, alpha, and beta bands during maintenance. These results suggest that the neural impact of spatial distraction is concentrated primarily during mnemonic maintenance.

## Data Availability

The raw data supporting the conclusions of this article will be made available by the authors, without undue reservation.
