# Supplementary material for: A multichannel MEG time–frequency analysis framework for detecting stage -specific effects of spatial distraction in visual-spatial working memory
Source: Front Neurosci. 2026 May 8;20:1844642. doi: 10.3389/fnins.2026.1844642 (PMC13195020; doi:10.3389/fnins.2026.1844642)
Supplement: Supplementary file 1 [file Table_1.docx]

Supplemental Table 1 Cluster-level statistics and effect sizes of distractor-related oscillatory clusters, with Bonferroni correction across frequency bands within predefined task epochs.

| Cluster_ID | Cluster-level  p value (uncorrected) | Bonferroni correction  significance | Within-subject effect size (Cohen’s $d_{z}$) |
| --- | --- | --- | --- |
| Encoding beta cluster #1 | 0.031 | No | -1.807 |
| Maintenance alpha cluster #1 | 0.004 | Yes | 1.476 |
| Maintenance beta cluster #1 | 0.001 | Yes | 1.168 |
| Maintenance delta cluster #1 | 0.018 | No | 1.159 |
| Maintenance delta cluster #2 | 0.024 | No | 1.023 |
| Maintenance theta cluster #1 | 0.0087 | Yes | 0.914 |

Bonferroni correction was applied across frequency bands within each predefined epoch. Direction of the condition effect (Distractor vs. No-distractor).
